# Degradation of Human PDZ-Proteins by Human Alphapapillomaviruses Represents an Evolutionary Adaptation to a Novel Cellular Niche

**DOI:** 10.1371/journal.ppat.1004980

**Published:** 2015-06-18

**Authors:** Koenraad Van Doorslaer, Rob DeSalle, Mark H. Einstein, Robert D. Burk

**Affiliations:** 1 Department of Microbiology & Immunology, Albert Einstein College of Medicine, Bronx, New York, New York, United States of America; 2 Sackler Institute of Comparative Genomics, American Museum of Natural History, New York, New York, United States of America; 3 Department of Obstetrics & Gynecology and Women’s Health, Albert Einstein Cancer Center, Albert Einstein College of Medicine, Bronx, New York, New York, United States of America; 4 Department of Pediatrics, Albert Einstein Cancer Center, Albert Einstein College of Medicine, Bronx, New York, New York, United States of America; Fred Hutchinson Cancer Research Center, UNITED STATES

## Abstract

In order to complete their life cycle, papillomaviruses have evolved to manipulate a plethora of cellular pathways. The products of the human *Alphapapillomavirus* E6 proteins specifically interact with and target PDZ containing proteins for degradation. This viral phenotype has been suggested to play a role in viral oncogenesis. To analyze the association of HPV E6 mediated PDZ-protein degradation with cervical oncogenesis, a high-throughput cell culture assay was developed. Degradation of an epitope tagged human MAGI1 isoform was visualized by immunoblot. The correlation between HPV E6-induced degradation of hMAGI1 and epidemiologically determined HPV oncogenicity was evaluated using a Bayesian approach within a phylogenetic context. All tested oncogenic types degraded the PDZ-containing protein hMAGI1d; however, E6 proteins isolated from several related albeit non-oncogenic viral types were equally efficient at degrading hMAGI1. The relationship between both traits (oncogenicity and PDZ degradation potential) is best explained by a model in which the potential to degrade PDZ proteins was acquired prior to the oncogenic phenotype. This analysis provides evidence that the ancestor of both oncogenic and non-oncogenic HPVs acquired the potential to degrade human PDZ-containing proteins. This suggests that HPV E6 directed degradation of PDZ-proteins represents an ancient ecological niche adaptation. Phylogenetic modeling indicates that this phenotype is not specifically correlated with oncogenic risk, but may act as an enabling phenotype. The role of PDZ protein degradation in HPV fitness and oncogenesis needs to be interpreted in the context of *Alphapapillomavirus* evolution.

## Author Summary

It is thought that the ability to degrade PDZ domain containing proteins is a hallmark of oncogenic papillomaviruses. However, since papillomaviruses did not evolve to be oncogenic, this hypothesis does not address the evolutionary importance of this phenotype. The present manuscript attempts to address whether HPV induced degradation of PDZ containing proteins is associated with oncogenic potential as determined by the clinical/epidemiological empirical cancer risk. Using Bayesian approaches to model trait evolution we show that it is highly unlikely for a virus to become oncogenic without first acquiring the ability to degrade PDZ proteins. Furthermore, the ability to degrade PDZ proteins allowed ancestral viruses to colonize a new cellular niche. However, in order to thrive in this new environment, these ancestral viruses had to acquire additional functions. We hypothesize that some of these additional phenotypes lead to oncogenicity. Importantly, our study illustrates the power of combining epidemiological, biochemical and evolutionary data with phylogenetic analysis in attempting to understand the relative role of specific pathogen phenotypes with host pathogenesis.

## Introduction

Papillomaviruses (PVs) are a diverse family of dsDNA viruses infecting most, if not all, amniotes. Based on nucleotide similarities, PVs are classified into genera identified by Greek letters. A genus is further divided into numbered species [1,2]. Persistent infection with specific human papillomaviruses (HPVs) has been shown to be necessary for the induction of cervical carcinoma [3,4]. All established oncogenic HPV types (OTs) belong to the genus *Alphapapillomavirus* [5]. Of note, phylogenetically, these oncogenic HPV types cluster into a so-called high-risk (HR) clade, indicating an evolutionary relationship between these viruses [5,6]. Importantly, available epidemiological data suggests that while some HPV types (e.g. HPV16) within this HR clade are strongly associated with cancer, others (e.g. HPV68) are only oncogenic in rare cases [7]. Throughout papillomavirus evolution PVs continuously adapted to new ecological niches on the host. This process selected for PVs with specific phenotypes needed to interact with changing cellular environments. It is highly unlikely that the ability to cause malignancy provided certain PVs with an evolutionary advantage. This suggests that the *Alphapapillomaviruses* acquired a particular combination of phenotypes while adapting to a specific ecological niche. In some viruses, the resulting cellular insult may inadvertently drive the infected host cell towards transformation. We previously postulated that use of phylogenetic, epidemiological and biochemical analyses would be essential for the identification of viral phenotypes specifically associated with oncogenicity [5,8,9,10].

Most papillomaviruses express at least 7 proteins, two of which—E6 and E7—have been demonstrated to be sufficient for oncogenesis. To date, the exact mechanisms by which these viral proteins cause cellular transformation is unknown. The viral E6 and E7 proteins interact with a diverse set of cellular pathways. Some of these interactions have been proposed to be unique to oncogenic viruses, while others appear to be shared by all investigated types [reviewed in 10,11,12,13,14,15,16].

It is well established that the E6 protein from specific HPVs targets PDZ containing proteins for degradation [17]. PDZ domains represent an abundant class of protein interaction modules that target specific motifs on partner proteins. PDZ containing proteins regulate multiple biological processes including differentiation and the maintenance of cellular polarity [17,18,19,20,21]. The interaction with PDZ containing proteins is dependent on a canonical class I PDZ binding motif (PBM) at the extreme C-terminal of the E6 protein [22]. Several E6 proteins interact with the PDZ-protein MAGI1 (a member of the membrane-associated guanylate kinase (MAGUK) protein family) [23], and MAGI1 is highly sensitive to degradation by E6 proteins [24,25]. However, only HPVs containing a type I PBM are able to degrade MAGI1 *in vitro*, and it has been stated that only oncogenic types contained such a motif [26], implying that interactions with PDZ containing proteins are critical for HPV-induced oncogenesis [27].

In the present paper, we provide evidence that all members of the HR-HPV clade (i.e., α-5, -6, -7, -9 and -11 species groups) indeed contain a class I PBM. Nevertheless, there does not appear to be a correlation between the presence of this motif and oncogenic classification. We also present data that all tested HR types, regardless of their oncogenic potential, degrade a human MAGI protein (hMAGI1d). Importantly, the design of our study (in which we tested E6 proteins from viruses representing all known species within the genus *Alphapapillomavirus*) allowed us to evaluate this phenotype from an evolutionary perspective. The derived evolutionary model demonstrates that the capability to degrade hMAGI1 entered the viral-host relationship prior to the oncogenic phenotype. In addition, while PDZ protein degradation is not sufficient for cancer development, the model suggests that it enabled the evolution of a phenotype associated with cell transformation. These data further support the notion that biological processes are best understood once their evolutionary origin is taken into account [28].

## Materials and Methods

### Ethics statement

The patient samples used in this study have been IRBB approved. (IRB number: 2009–274; Approval date: 07/08/2009; Title: Epigenetic Profiling of Cervical Cancer: A Pilot Study.)

### Cell lines and constructs

The C-33A cell line is an HPV negative cervical cancer cell line expressing a mutant p53 protein [29,30] and was obtained from the ATCC (Manassas, VA, USA). The C-33A cells were maintained in Dulbecco’s modified Eagle’s medium (DMEM) with 10% fetal bovine serum (FBS). Western blot analysis

C-33A cells were plated in 6-well plates (800,000 cells/well) and transfected with 1400 ng pQCXIN-HA-hMAGI1d, 600 ng of each pQCXIN based E6 expression vector and 100 ng pSUPER-GFP (transfection control) using lipofectamine LTX (Invitrogen) according to the manufacturer’s instructions. The cells were lysed in RIPA buffer (Millipore, Billerica, MA, USA), containing appropriate protease inhibitors. Equal amounts of protein lysates (10 ug) were separated by SDS-PAGE. Western blot membranes were probed with antibodies recognizing the HA-tag (Cell Signaling, Danvers, MA, USA), eGFP (Cell Signaling) or actin (Sigma).

### Epidemiological classification and HPV type selection

Epidemiological classification of HPVs is based on a recent review of available world-wide reported data as conducted by an expert panel [7]. E6 proteins were selected to include at least one representative for each species within the genus *Alphapapillomavirus*. In addition, closely related viruses with differences in epidemiological classification were selected. The isolation and cloning of E6 ORFs has been described previously [8,9].

### Phylogenetic tree construction

**Fig 1:**
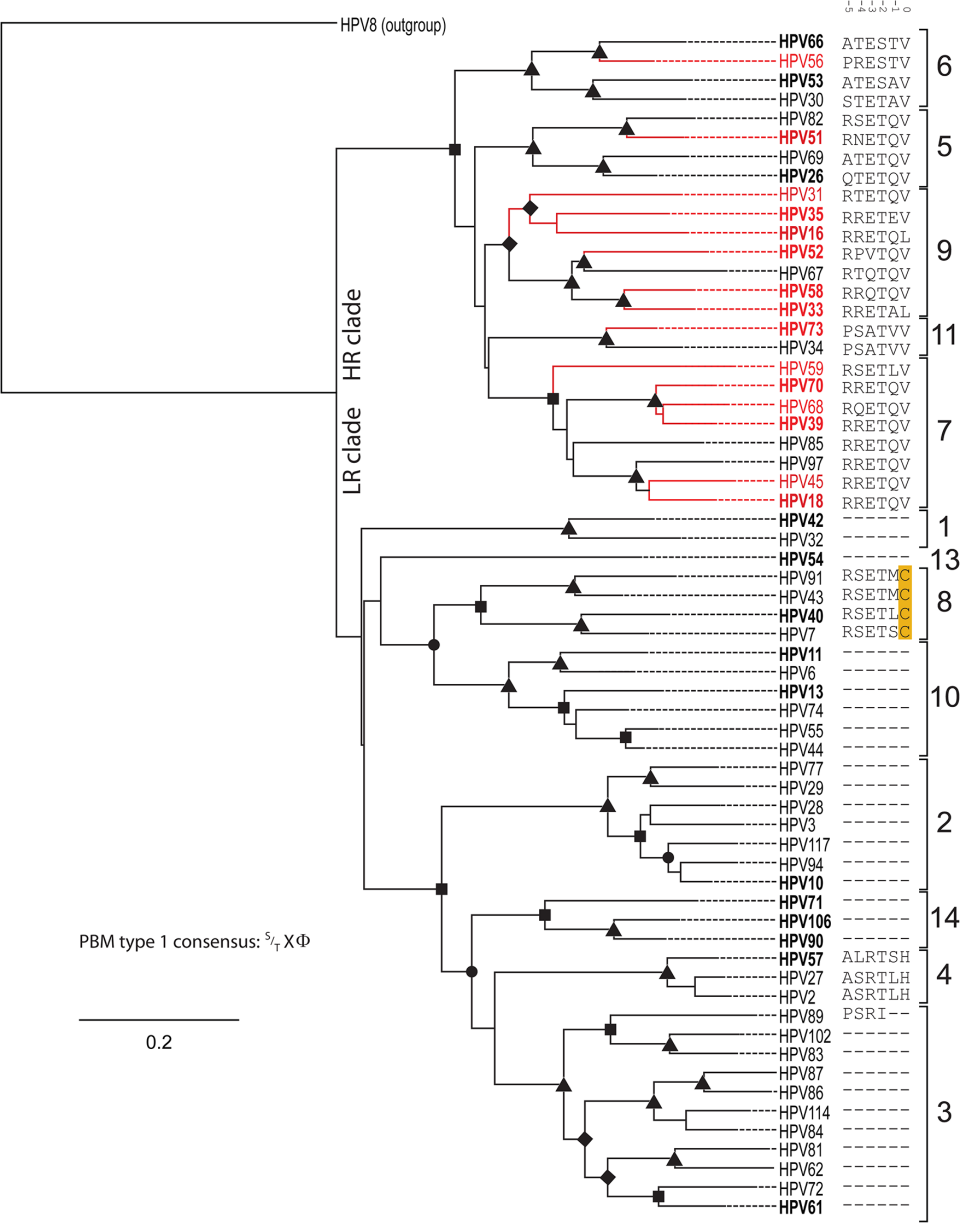
Presence of C-terminal PDZ binding motif is correlated with phylogenetic classification. The Bayesian phylogenetic tree is constructed based on the E6 nucleotide sequences of all known human *Alphapapillomaviruses*. HPV8, a *Betapapillomaviruses* was used to root the tree. The solid branches indicate the optimized branch lengths. Dotted lines were added to the branches to facilitate visual inspection of the tree. Epidemiological classification divides the tree into two main clades (“high-risk” vs.”low-risk”). Oncogenic papillomavirus types are colored red (classification based on [7]). Posterior probability values are indicated using symbols at the nodes (triangle = 1; rectangle > 0.95; circle>0.90; diamond >0.80). The numbers to the right indicate the different viral species within the genus *Alphapapillomaviruses*. The sequence of the six C-terminal residues constituting a putative type 1 PBM is indicated following the virus name. A dash (“-“) indicates the absence of a residue at that position. Numbers above the sequences allow for easy identification of landmark residues as in [42]. Viruses in **Bold** were selected for the *in vivo* analyses.

E6 sequences representing all viral types within the genus *Alphapapillomavirus* were downloaded from the PaVE database (http://pave.niaid.nih.gov/#home)[31]. The DNA sequences were translated to amino acids and aligned using MAFFT (the L-INS-I algorithm was used) [32], and the corresponding nucleotide coding regions were aligned within the Seaview program [33]. Bayesian tree reconstruction was performed using MrBayes [34,35] while implementing the GTR+I+G model (as selected by Jmodeltest2 [36,37]; S1 Table). To ensure the most efficient Bayesain analysis the convergence of the Markov chain Monte Carlo (MCMC) chains was explored graphically using AWTY [38]. The final posterior sample of trees is summarized as a majority rule consensus tree in Fig 1.

### Analysis of trait evolution

The viral traits under study (MAGI1 degradation and oncogenic potential) were coded as discrete presence/absence variables. Clades for which no epidemiological and/or biochemical data were available were coded as missing, and were considered ambiguous by the program. The computer program BayesDiscrete [39] (available from http://www.evolution.rdg.ac.uk) was used to detect evidence of correlated evolution between discrete traits. In order to account for phylogenetic uncertainty during the tree building process, 500 trees were randomly sampled from the post-burn-in posterior sample of MrBayes trees. The reversible jump MCMC analysis was run for 1x10^9^ iterations. After a burn-in of 1x10^6^ iterations, the chains were sampled every 10,000^th^ iteration. The alpha and beta parameters of a Gamma distribution were seeded from uniform distributions on the interval 0 to 2. To confirm the robustness of the results, the analysis was performed three independent times. Bayes factors were used to select between models [39]. Traditionally, a log Bayes Factor greater than 30 is considered as positive evidence for the model being tested.

## Results

### PDZ domain binding motifs are evolutionary conserved in members of the high-risk clade within the genus *Alphapapillomavirus*

In an attempt to understand differences in oncogenic potential, researchers have traditionally compared viral phenotypes between the prototypical HR and LR types (HPV16/HPV18 vs. HPV6/HPV11) [13]. However, these viruses infect different anatomical niches on the human body and are separated by approximately 30 million years of evolutionary divergence, confounding simple comparison [10]. Phylogenetic analysis (Fig 1), clustered the members of the genus *Alphapapillomvirus* into two main clades [10]. Epidemiological evidence has shown that only a subset of the viruses in this HR clade (highlighted in red) is actually associated with cervical cancer [7,40,41]. The alignment of all known *Alphapapillomavirus* E6 proteins (see panel to the right of Fig 1; 6 C-terminal residues are shown) illustrates that the presence of a canonical type 1 PBM (S/TXΦ where Φ indicates any hydrophobic residue [42]) represents a synapomorphy of the high-risk papillomaviruses. In other words, all HR viruses, independent of associated oncogenic risk have a PDZ interacting domain. While most viruses in the LR clade do not contain comparable residues, HPV types belonging to the *Alphapapillomavirus* 8 species contain an E6 protein with a similar motif. The sequence analysis suggests that these alpha-8 E6 proteins may be able to target PDZ containing proteins for degradation. Thus, the E6 proteins from several non-oncogenic viruses contain putative PBMs, suggesting that the presence of a type 1 PBM does not allow for dichotomization between oncogenic and non-oncogenic HPV types. However, the presence of a sequence motif does not establish that these E6 proteins actually target PDZ proteins for degradation. Therefore, we examined the effects of E6 protein expression on steady state levels of a human PDZ-protein *in vivo*.

### The E6 proteins from high-risk papillomaviruses induce degradation of a PDZ containing protein

**Fig 2:**
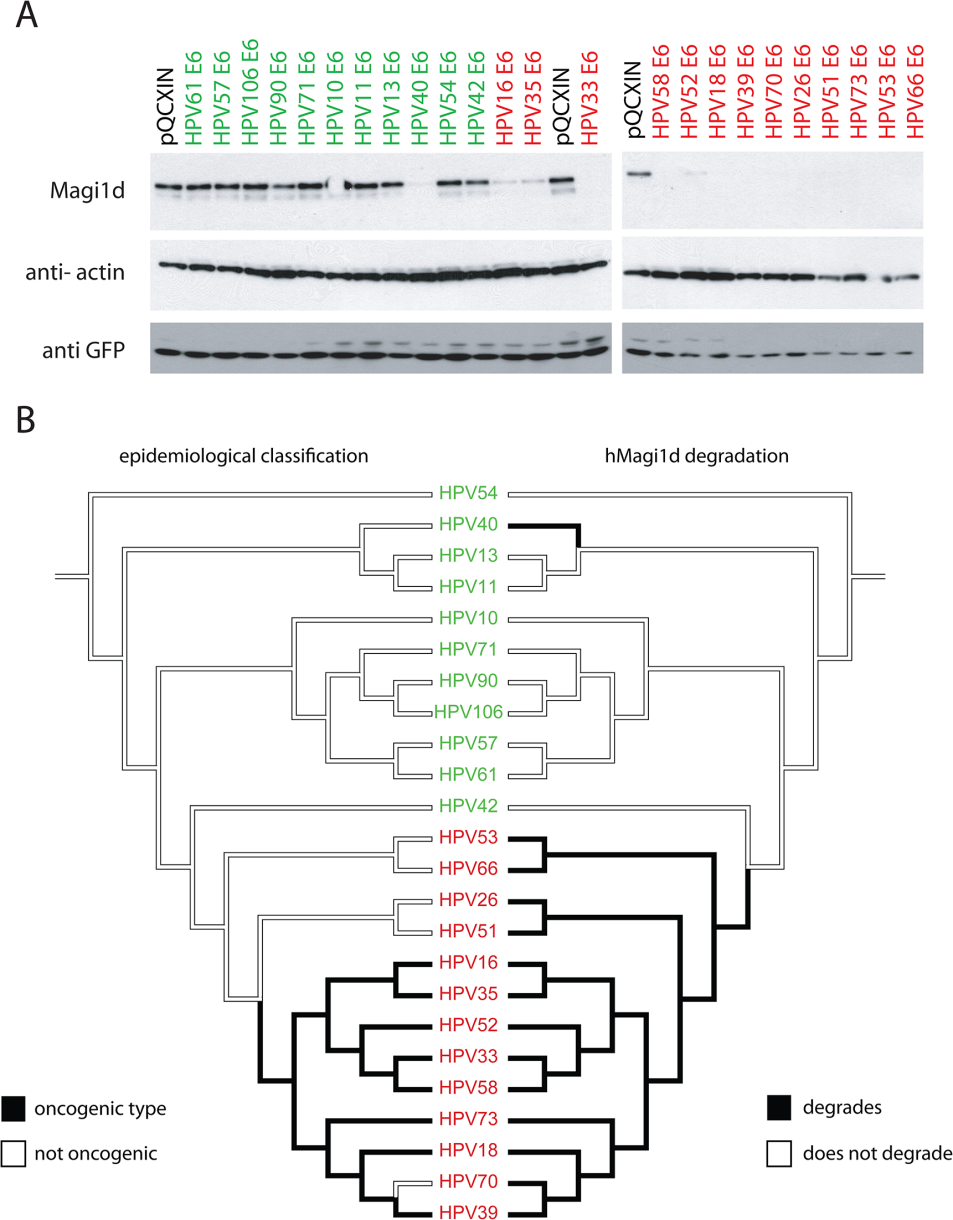
All members of the phylogenetic high-risk clade degrade human MAGI1d. (A) C-33A cells were transfected with 24 different E6 proteins covering the known evolutionary spectrum within the *Alphapapillomavirus* genus. The western blot shows a representative experiment. GFP was probed as a transfection control indicating equal transfection. This figure shows that all High-Risk types (highlighted in red) target hMAGI1d for degradation. The pQCXIN vector was used as control. (B) Mirror trees comparing the epidemiological (left) and PDZ-protein degradation (right) phenotypes on the E6 based phylogeny. The viral names are colored according to phylogenetic classification. High-risk viruses are colored in red, while LR viruses are colored green. The branches of the tree are shaded according to the state of each character under investigation.

In order to test whether different E6 proteins affect the steady state levels of a human PDZ protein, a novel isoform of MAGI was cloned from cervical cells (S1 Methods). We tested the effects of co-expressing HPV E6 proteins with the PDZ containing human protein hMAGI1d (Fig 2A). The figure shows a representative gel of the obtained results. These data demonstrate that all members of the HR-clade (highlighted in red) degrade hMAGI1d when compared to the empty vector control (pQCXIN). In addition, HPV40 E6, a representative of the alpha-8 species group, a viral type embedded in the LR-clade also degraded hMAGI1d in this system (Fig 2 and S1 Fig). Thus, the alpha-8 PBM (Fig 1) is associated with hMAGI1d degradation. It has been shown that the HPV E6 proteins target PDZ-proteins for proteasomal degradation [43,44]. We confirmed that the tested E6 proteins (including HPV40 E6) degrade human MAGI1d in a proteasome dependent manner (S1 Fig). HPV oncogenic potential and MAGI1d degradation is contrasted using mirrored trees in Fig 2B. The left tree is shaded according to epidemiological classification, while the tree on the right indicates the ability of HPV types to degrade MAGI1 as shown in Fig 2A. While this data indicates incoherence between both PDZ degradation and oncogenic activity, this hypothesis is formerly tested in the next paragraph.

### hMAGI1d degradation by HPV E6 proteins represents an enabling phenotype towards oncogenicity

**Fig 3:**
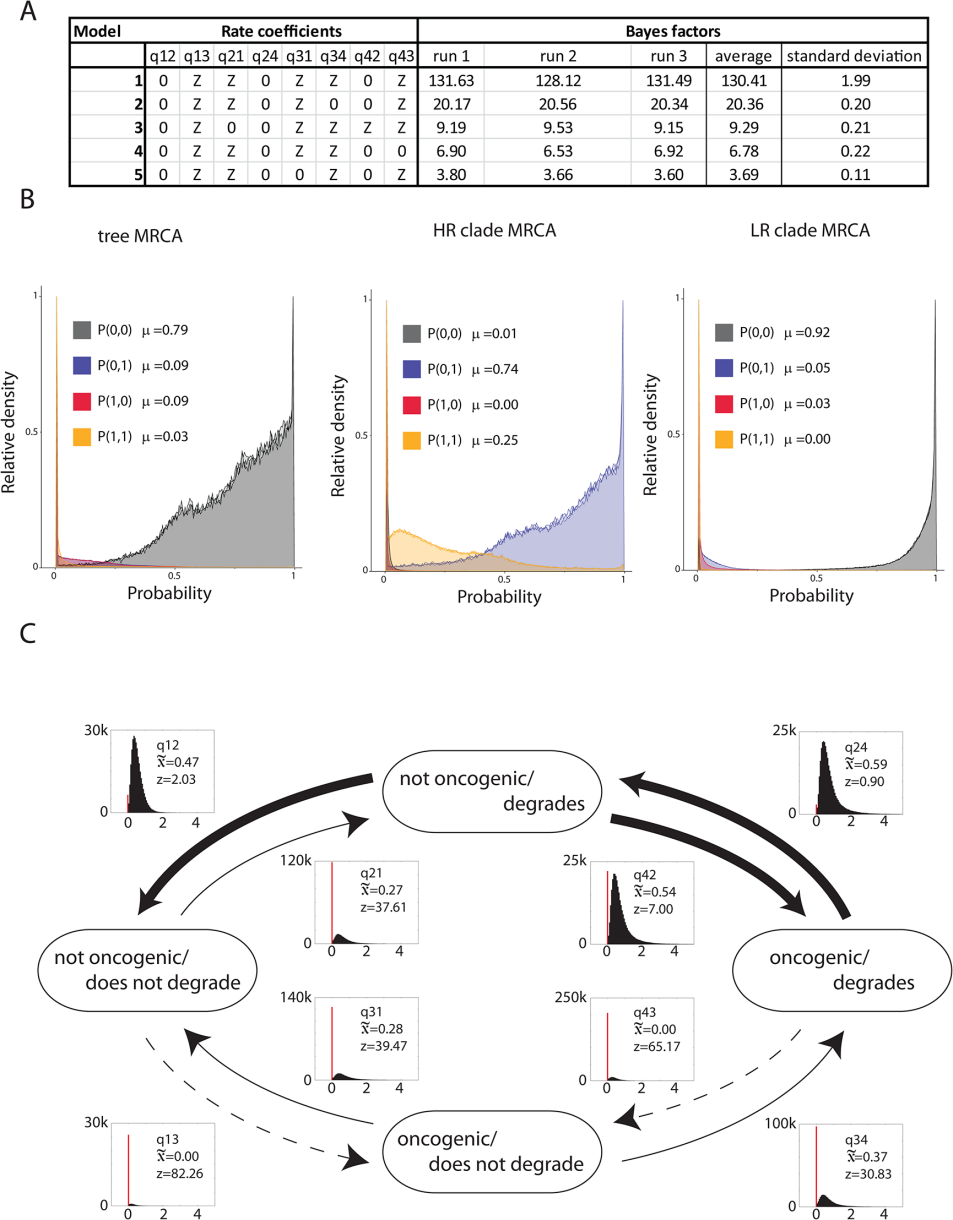
Degradation of PDZ proteins is an enabling phenotype towards oncogenicity. (A) Table of top five models as selected by the RJMCMC analysis. The RJMCMC was run three independent times to ensure that the reproducibility of the model analysis. Through comparing the ratio of posterior to prior odds (see materials and methods) we obtain Bayes Factors to support the choice of a specific model. The selected model has a Bayes Factor of 130.41 (st.dev. = 1.99) suggesting decisive evidence in favor of this model. (B) Ancestral phenotype reconstruction at three important nodes of the *Alphapapillomavirus* phylogeny. The graphs show the estimated marginal probability density plot for the common MRCA, the high-risk MRCA or the low-risk MRCA. The analysis suggests that the MRCA of the high-risk viruses acquired the ability to degrade PDZ-containing protein, but was likely not an oncogenic virus. (C) Estimated instantaneous rates of change between different combinations of viral phenotypes based on the RJMCMC analysis. Histograms show the posterior distribution of estimated values of the rate parameters. The red bars indicate the fraction of samples in which each rate is estimated to be zero. Arrow width is proportional to the median of these estimated rates. Z indicates the percentage of samples in which each rate parameter is estimated as zero. Consistent with the hypothesis that the ability to degrade PDZ containing proteins is an enabling phenotype, rates associated with oncogenic ability independent of PDZ protein interaction are often estimated as zero (i.e. they do not occur), and are thus represented as dotted line.

The main goal of this study was to test whether degradation of PDZ containing proteins was associated with oncogenic potential as determined by the clinical/epidemiological empirical cancer risk [7]. The biochemical degradation of PDZ-proteins and viral oncogenic potential were coded as discrete variables, and tested to determine whether these pairs of discrete binary traits evolved together. Briefly, the test of correlated evolution, as implemented within the BayesTraits package [39], compares the fit of two models of evolution. In the independent model both traits are allowed to evolve independently on the tree. The dependent model requires that both traits evolve in a correlated fashion. In order to incorporate phylogenetic uncertainty we integrated the analysis over 500 trees randomly selected from the posterior distribution. We employed a reversible-jump (RJ) MCMC methodology [39]. The RJ-MCMC has the advantage that it travels through the posterior tree sample and tests *all possible* models of evolution in proportion to the likelihood of each individual model. This method provides a posterior sample (n = 100,000) of models with their associated transition rates. We obtain support for each model through the calculation of Bayes Factors based on the frequency with which each model occurs in the posterior sample compared to the prior expectation of observing that model (i.e., the ratio of posterior to prior odds) [45]. Bayes Factors are interpreted on a heuristic scale. Traditionally, Bayes Factors greater than 30 are considered very strong evidence in favor of one model. To ensure reproducibility of the RJ-MCMC analysis, the analysis was performed three independent times. Fig 3A shows the top 5 models selected by the RJ-MCMC approach. The selected model (“0ZZ0ZZ0Z”; Fig 3A) has a Bayes Factor of 130.41 (+/- 1.99) demonstrating strong support for this model. We also reconstructed the ancestral combination of phenotypes at important nodes of the phylogenetic tree. This suggested that the most recent common ancestor (MRCA) of all high and low-risk *Alphapapillomaviruses* did not degrade PDZ proteins and was not oncogenic (probability P(0,0) = 0.79; Fig 3B, left panel). Likewise, the MRCA of the LR clade did not degrade PDZ proteins. However, the ancestor of the extant HR viruses acquired the ability to degrade PDZ proteins. Importantly, the analysis suggests that this ancestral HR virus would not have been oncogenic (P(0,1) = 0.74; Fig 3B, middle panel).

Fig 3C shows the estimated instantaneous rates change between the four different phenotype combinations. The selected model suggests that the ancestor of the HR clade acquired the ability to degrade PDZ proteins, which is associated with a relatively low probability, likely reflecting the fact that a new interaction motif had to be created. This combination of phenotypes (able to degrade PDZ proteins while not oncogenic) appears to be highly unstable, and suggests that the ability to degrade certain PDZ proteins resulted in sub-optimal viral fitness. Under these assumptions, the model predicts two equally probable scenarios. In the first scenario, the progeny viruses would lose the capacity to degrade PDZ containing proteins. Since no extant members of the HR clade lost the ability to degrade PDZ proteins, we hypothesize that such viruses were unable to colonize the available niche and went extinct. The second scenario proposes that the ancestors of the extant HR viruses were able to colonize and thrive in a new ecological environment. It is likely that these viruses had to evolve ways to cope with the cellular milieu following the loss of PDZ containing proteins. Furthermore, the model predicts that it is highly unlikely to become oncogenic without degrading MAGI1. Taken together the evolutionary data suggests that the ability to degrade PDZ proteins represents an enabling phenotype that had to be acquired prior to further adaptation. Some of these additionally adopted phenotypes inadvertently resulted in cellular deregulation, transformation and cancer.

## Discussion

In the present study we tested the ability of E6 proteins from different HPV types to degrade PDZ containing proteins. We used evolutionary hypothesis testing to reconstruct the plausible evolutionary events involved in E6-induced PDZ-protein degradation and HPV type cancer risk. These analyses indicated that the biochemical phenotype was not associated with the cancer risk, but rather with the evolution of extant viral types.

Due to their small genomic size, the complete genomic sequence of over 200 papillomaviruses has been characterized [31]. Importantly, the empirically derived contribution of most HPVs to (cervical) cancer has been well documented. While (cervical) carcinogenicity of the HR-HPV types likely varies in strength along a continuum without clear demarcation, from extremely strong (e.g. HPV16) to probably carcinogenic in rare situations (e.g. HPV 68), evidence suggests that only a small subset of viruses is responsible for virtually all cancers Of note, viruses closely related to these oncogenic HPV types are significantly less common in cancer, despite the fact that their overall prevalence is comparable. Suggesting that these related viruses are implicitly less carcinogenic. Taking into account some of the limitations associated with epidemiological classification, the present study only considered viruses as oncogenic if sufficient epidemiological evidence was available based on analysis by experts [41].

The analysis of biochemical assays in light of the available genomic information and epidemiological classification has allowed us to predict which viral phenotypes may be pathogenic and begin to explain why only a subset of these viruses is truly oncogenic. The use of evolutionary information has been valuable to interpret the relationship between HPV type specific phenotypes and HPV oncogenicity [8,9].

While developing the *in vivo* assay, we cloned and sequenced a novel isoform of the MAGI1 family, hMAGI1d. We provide evidence that this isoform is expressed in cervical cell lines and primary cervical tumors. We confirmed that, as was previously reported for other MAGI proteins, HPV16 E6 dramatically affects the steady state levels of exogenously expressed human MAGI1d [43] and that this reaction is dependent on the proteasome. Since hMAGI1d appears to be degraded by HPV E6 proteins in a manner similar to what has been reported for other PDZ containing proteins we used this human isoform as a representative member of the PDZ-protein family. Previous studies have shown that specific residues within the PBM and/or certain post-translational modifications may result in each E6 protein having a specific PDZ protein interactome [23,46,47,48]. However, the goal of this study was to relate the overall ability to degrade PDZ containing substrates to oncogenic potential. It was previously shown that most E6 proteins interact with MAGI1 with nano-molar affinity [49]. Therefore, by comparing the steady state levels of hMAGI1d, and not the kinetics of degradation, this approach allows for the representation of the degradation phenotype as a discrete variable.

The analysis of 24 tested viral E6 proteins showed that all members of the HR-clade, independent of oncogenic risk-classification were capable of degrading hMAGI1d. In addition, we provide evidence that the E6 protein of HPV40, a member of the alpha-8 species, contains a functional type 1 PBM. While HPV E6 proteins appear to prefer a Val or Leu residue, any hydrophobic residue can allow for interaction with PDZ containing proteins [42]. Indeed, the PBM found on the HBV core protein contains a terminal cysteine [50,51]. The observation that the members of the alpha-8 clade are the only low-risk viruses that encode E6 proteins with a functional PBM may be the result of convergent evolution. This is supported by the presence of a divergent cysteine (as opposed to the canonical valine or leucine). Importantly, this implies that (some) members of the low-risk clade have the inherent ability to degrade cellular targets. Indeed, we have previously shown that the E6 protein derived from the low-risk virus HPV71 is able to degrade p53 [8]. Furthermore, when the HPV18 PBM was grafted onto HPV11 E6, this chimeric protein was able to degrade cellular PDZ proteins [52]. This supports the notion that the ability to degrade cellular protein is inherently shared by all *Alphapapillomaviruses* [52].

We used evolutionary trait analysis to integrate biochemical, epidemiological and evolutionary data. This approach allowed us to model how two viral phenotypes (the ability to degrade PDZ containing proteins and viral oncogenic potential) evolved. Importantly, we were able to estimate the order of evolutionary events in the emergence of these traits in HPVs. Since all tested members of the High-risk clade are able to degrade hMAGI1d, the most parsimonious explanation suggests that the putative ancestor of this clade was likewise a degrader. Indeed, the trait analysis favors the hypothesis that the ancestor of the extant high-risk viruses gained the ability to degrade PDZ-containing proteins. Notably, this putative ancestral virus was likely not oncogenic. A key result indicates that the ability to degrade PDZ-proteins is a highly unstable phenotype. This suggests that even though the initial acquisition of a type I PBM lowered viral fitness, it may have allowed for the colonization of a new ecological niche. Furthermore, since the acquisition of PDZ degradation did not coincide with the ability of viruses to cause cancer, the oncogenic types must have acquired additional phenotype(s) that explain their association with human cancer. We have previously reported that the ability to increase cellular hTERT, the protein subunit of telomerase, shows strong association with epidemiological classification [9]. Importantly, since the oncogenic viruses do not form a monophyletic clade, oncogenicity may represent convergent evolution. Alternatively, the non-oncogenic types within the high-risk-clade may have reduced penetrance of the oncogenic phenotype(s), thereby making them less oncogenic.

The present study provides evidence that all members of the High-Risk clade are able to target PDZ containing proteins for degradation. Phosphorylation of the PBM by cellular kinases modulates the ability of viral E6 proteins to recognize PDZ containing substrates. The observation that evolutionarily related E6 proteins are substrates of divergent kinases [48], suggests that the acquisition of a PBM predates the (convergent) evolution of regulation through post-translational modifications.

By analyzing extant E6 proteins, a previous study did not find evidence for a strict correlation between *in vitro* PDZ protein degradation and oncogenicity [26]. However, in order to fully understand the importance of E6/PDZ-protein interaction and degradation in the malignant process, it is important to analyze this phenotype in relationship to the evolutionary history of these viruses. By taking evolutionary relationships into account we propose a model in which the ability to degrade PDZ proteins allowed for the colonization of a new cellular niche (e.g the cervical transformation zone).

A role for PDZ protein degradation during the viral lifecycle is supported by the observation that mutants of HPV31 unable to interact with PDZ proteins are less fit compared to wild type viruses. Specifically, cellular proliferation, viral copy number control and other early viral functions were affected [20]. A PBM is required for long-term replication of the viral genome. Interestingly, this requirement is alleviated when p53 is removed from the cell using shRNA [53]. While, all tested members of the HR-clade were capable of degrading both p53 and hMAGI1d [8], low-risk viruses do not show clear correlation between both phenotypes. HPV71, a virus previously shown to degrade p53 [8], did not affect the steady state levels of MAGI1d. Inversely, HPV40 does not degrade p53. Therefore, the association between the ability to degrade p53 and PDZ-proteins may be less clear than previously suggested [43].

In conclusion, the ability of E6 proteins to interact with PDZ proteins allowed papillomaviruses to colonize a new ecosystem in the host. However, in order to thrive within this new environment the virus evolved additional ways to usurp the host cells’ machinery. Through interfering with normal differentiation and cell cycle control pathways, long term persistent infection may prime the cell for malignant transformation. The acquisition of hTERT promoter activation (and/or other interactions) by the oncogenic viruses might begin to explain why these viruses are associated with significantly higher cancer rates compared to non-oncogenic types [9].

Finally, this study highlights the importance of combining epidemiological, biochemical and evolutionary data with phylogenetic analysis in attempting to understand the relative role of specific viral phenotypes with host pathogenesis.

## Supporting Information

S1 Fig HPV40 E6 mediated degradation of hMAGI1d is dependent on the proteasome. C33A cells were transfected with the indicated plasmids. Twenty-four hrs after transfection, the cells were either treated with MG132 (10 uM final concentration) or DMSO for 16 hrs. Proteasome inhibition restores the levels of hMAGI1d in cells co-expressing HPV16 and HPV40 E6.
(PDF)

S2 Fig Cervical cells express a novel, shorter isoform of MAGI1. (A) intron-exon boundaries of the MAGI1 gene. The complete MAGI1 gene was downloaded using the UCSD genome browser (http://genome.ucsc.edu/). The cDNAs for the respective splice variants were downloaded from NCBI and aligned to the complete genomic sequence using Spidey (http://www.ncbi.nlm.nih.gov/spidey/index.html). (B) The main motifs identified in hMAGI1c (top) and hMAGI1d (bottom). hMAGI1d does not contain the 5^th^ PDZ domain (PDZ4). Positioning of domains is according to SMART [54,55]. In addition to PDZ domains both proteins contain two WW domains (red oval) and a guanylate kinase domain (orange hexagon). (C) RT-PCR of hMAGI1d from cervical samples. Total RNA was extracted from the C-33A cell line and 4 patient samples (CCx1-4). RNA was converted to cDNA (with or without RT enzyme), and cDNA was amplified (see materials and methods). Arrowheads indicate the position of hMAGI1d (3900 bp). Asterisks (*) indicate non-specific PCR amplicons. A DNA ladder is shown in the left lane, labeled MM. Addition of reverse transcriptase (RT) is indicated at the top of the gel.
(PDF)

S3 Fig Location of PCR primers used to screen for the presence of hMAGI1d. Pairwise sequence alignment of hMAGI1c and hMAGI1d. Numbers on the right indicate the nucleotide position in the respective cDNA. Dashes indicate the absence of sequence in hMAGI1d. Primers used for PCR amplification are indicated.
(PDF)

S1 Table Results of the JModeltest run. Models are sorted according to the AIC criteria. The GTR+I+G model of evolution was selected.
(XLSX)

S2 Table Primers used in the cloning and screening of hMAGI1d. (XLSX)

S1 Methods Methods describing the cloning of a novel human MAGI1 isoform. (DOCX)
