# Supplementary figures and images for: Degradation of Human PDZ-Proteins by Human Alphapapillomaviruses Represents an Evolutionary Adaptation to a Novel Cellular Niche
Source: PLoS Pathog. 2015 Jun 18;11(6):e1004980. doi: 10.1371/journal.ppat.1004980 (PMC4472669; doi:10.1371/journal.ppat.1004980)

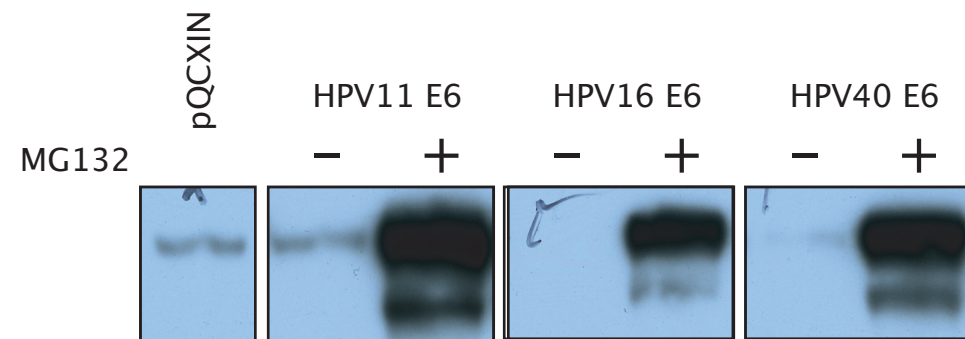

Supplement: S1 Fig — C33A cells were transfected with the indicated plasmids. Twenty-four hrs after transfection, the cells were either treated with MG132 (10 uM final concentration) or DMSO for 16 hrs. Proteasome inhibition restores the levels of hMAGI1d in cells co-expressing HPV16 and HPV40 E6. (PDF) [file ppat.1004980.s001.pdf]

A

chr 3 (545204-682457)

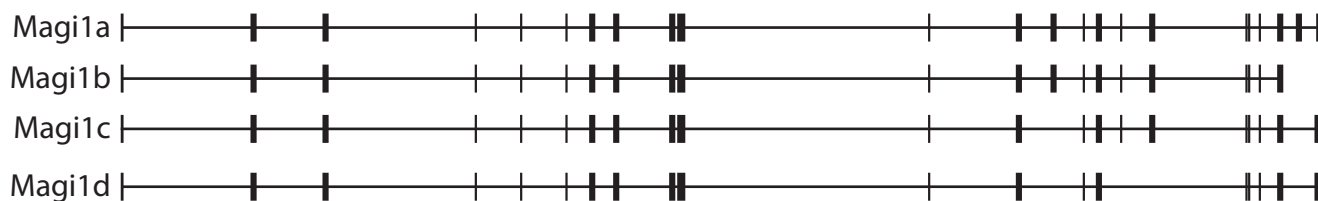

B

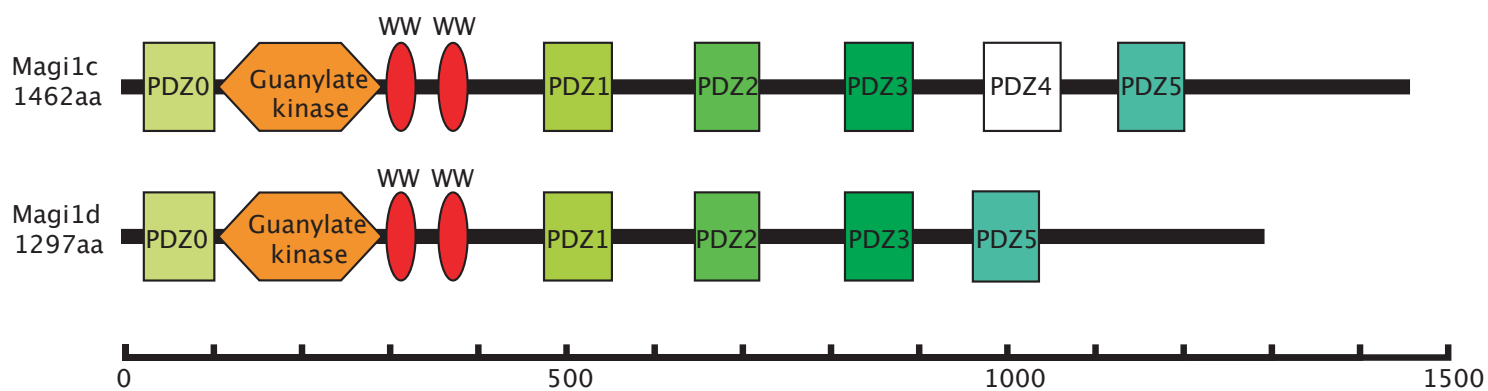

C

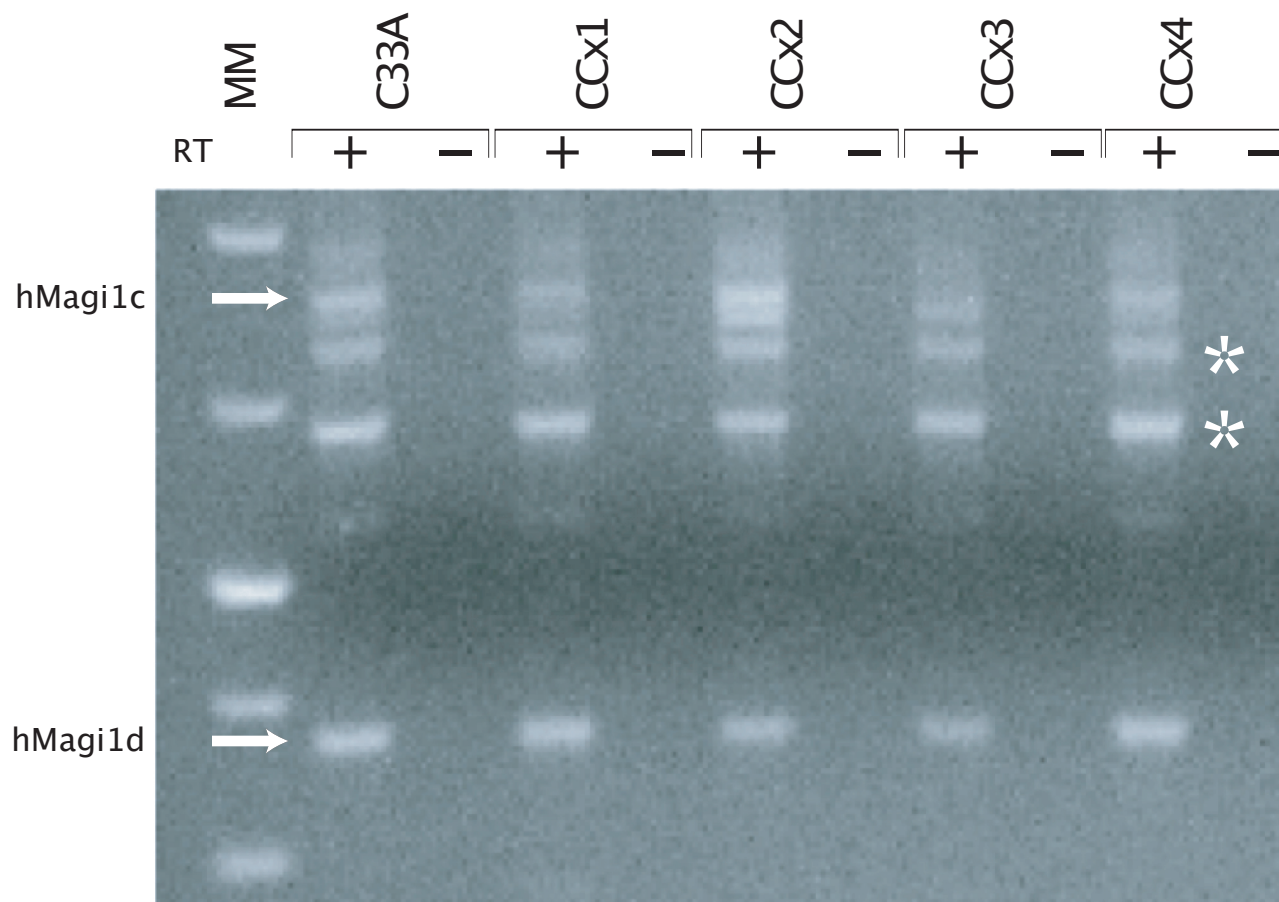

Supplement: S2 Fig — (A) intron-exon boundaries of the MAGI1 gene. The complete MAGI1 gene was downloaded using the UCSD genome browser (http://genome.ucsc.edu/). The cDNAs for the respective splice variants were downloaded from NCBI and aligned to the complete genomic sequence using Spidey (http://www.ncbi.nlm.nih.gov/spidey/index.html). (B) The main motifs identified in hMAGI1c (top) and hMAGI1d (bottom). hMAGI1d does not contain the 5th PDZ domain (PDZ4). Positioning of domains is according to SMART [54,55]. In addition to PDZ domains both proteins contain two WW domains (red oval) and a guanylate kinase domain (orange hexagon). (C) RT-PCR of hMAGI1d from cervical samples. Total RNA was extracted from the C-33A cell line and 4 patient samples (CCx1-4). RNA was converted to cDNA (with or without RT enzyme), and cDNA was amplified (see materials and methods). Arrowheads indicate the position of hMAGI1d (3900 bp). Asterisks (*) indicate non-specific PCR amplicons. A DNA ladder is shown in the left lane, labeled MM. Addition of reverse transcriptase (RT) is indicated at the top of the gel. (PDF) [file ppat.1004980.s002.pdf]
