# Supplementary material for: Degradation of Human PDZ-Proteins by Human Alphapapillomaviruses Represents an Evolutionary Adaptation to a Novel Cellular Niche
Source: PLoS Pathog. 2015 Jun 18;11(6):e1004980. doi: 10.1371/journal.ppat.1004980 (PMC4472669; doi:10.1371/journal.ppat.1004980)
Supplement: S3 Fig — Pairwise sequence alignment of hMAGI1c and hMAGI1d. Numbers on the right indicate the nucleotide position in the respective cDNA. Dashes indicate the absence of sequence in hMAGI1d. Primers used for PCR amplification are indicated. (PDF) [file ppat.1004980.s003.pdf]

supplemental figure 2

```
Magi1c    2401 ATGTATGAAAACCGACTTCCAGATTACCAGGAACAGGACATCTTCCTCTGGAGAAAAGAGACTGGATTTGGATTTAGGATTCTGGGTGGAAATGAACCAG   2500
Magi1d    2401 ATGTATGAAAACCGACTTCCAGATTACCAGGAACAGGACATCTTCCTCTGGAGAAAAGAGACTGGATTTGGATTTAGGATTCTGGGTGGAAATGAACCAG   2500
                                         Magi1-KVD007

Magi1c    2501 GGGAACCTATTTATATTGGTCACATCGTACCACTGGGTGCTGCTGATACTGACGGCCGCCTGAGGTCTGGAGATGAATTAATCTGTGTGGATGGGACGCC   2600
Magi1d    2501 GGGAACCTATTTATATTGGTCACATCGTACCACTGGGTGCTGCTGATACTGACGGCCGCCTGAGGTCTGGAGATGAATTAATCTGTGTGGATGGGACGCC   2600

Magi1c    2601 AGTAATTGGAAAATCACACCAGCTTGTGGTCCAGCTTATGCAACAAGCTGCCAAGCAAGGCCACGTCAATCTCACGGTGCGGCGTAAAGTGGTTTTTGCG   2700
Magi1d    2601 AGTAATTGGAAAATCACACCAGCTTGTGGTCCAGCTTATGCAACAAGCTGCCAAGCAAGGCCACGTCAATCTCACGGTGCGGCGTAAAGTGGTTTTTGCG   2700

Magi1c    2701 GTGCCCAAAACCGAGAACGAGGTGCCCTCGCCAGCCTCCTCTCATCACAGTAGCAACCAGCCGGCCTCGCTGACAGAAGAGAAGCGCACTCCGCAGGGCA   2800
Magi1d    2701 ----------------------------------------------------------------------------------------------------   2700

Magi1c    2801 GCCAGAACTCGCTGAACACGGTGAGCTCGGGCAGCGGCAGCACCAGCGGCATCGGCAGTGGCGGCGGCGGGGGCAGCGGCGTGGTCAGCACCGTGGTGCA   2900
Magi1d    2701 ----------------------------------------------------------------------------------------------------   2700

Magi1c    2901 GCCCTACGACGTGGAGATCCGGCGCGGGGAGAACGAGGGCTTCGGCTTCGTCATCGTGTCCTCGGTGAGCAGGCCCGAAGCAGGCACGACCTTTGGCAAT   3000
Magi1d    2701 ----------------------------------------------------------------------------------------------------   2700

Magi1c    3001 GCATGTGTGGCTATGCCTCACAAAATAGGTCGGATTATTGAGGGGAGCCCTGCTGACCGCTGTGGCAAGCTGAAAGTAGGAGACCGGATCTTGGCAGTAA   3100
Magi1d    2701 ----------------------------------------------------------------------------------------------------   2700

Magi1c    3101 ATGGATGTTCCATCACCAACAAATCCCATTCAGACATTGTGAACCTAATCAAGGAAGCGGGAAACACAGTTACCCTCCGCATCATTCCTGGGGATGAGTC   3200
Magi1d    2701 -----------------------------------------------------------------------------------------------GAGTC   2705

Magi1c    3201 CTCGAATGCCACCTTGCTGACCAATGCAGAGAAGATTGCCACCATCACCACCACACACACCCCTTCTCAGCAAGGGACCCAGGAGACAAGGAATACCACC   3300
Magi1d    2706 CTCGAATGCCACCTTGCTGACCAATGCAGAGAAGATTGCCACCATCACCACCACACACACCCCTTCTCAGCAAGGGACCCAGGAGACAAGGAATACCACC   2805
                                                                                            Magi1-KVD008

Magi1c    3301 AAACCAAAGCAGGAATCTCAATTTGAGTTCAAAGCACCCCAAGCAACACA   3350
Magi1d    2806 AAACCAAAGCAGGAATCTCAATTTGAGTTCAAAGCACCCCAAGCAACACA   2855
```
