# Supplementary material for: Degradation of Human PDZ-Proteins by Human Alphapapillomaviruses Represents an Evolutionary Adaptation to a Novel Cellular Niche
Source: PLoS Pathog. 2015 Jun 18;11(6):e1004980. doi: 10.1371/journal.ppat.1004980 (PMC4472669; doi:10.1371/journal.ppat.1004980)
Supplement: S1 Methods — (DOCX) [file ppat.1004980.s006.docx]

### Cloning of a human MAGI1 isoform

To clone human MAGI1 mRNA, primers flanking the predicted MAGI1c gene were designed to incorporate an N-terminal HA-tag and restriction enzyme sites (primer sequences are available in table S2). Total RNA was extracted from C-33A cells (RNaequos4PCR kit; Applied Biosystems, Carlsbad, CA, USA) and converted into cDNA as described below. A human MAGI1 mRNA was amplified and cloned into a pQCXIN vector. All constructs and PCR fragments were confirmed by Sanger sequencing performed at the Einstein sequencing facility. The cDNA sequence of hMAGI1d was deposited in GenBank (accession number KC170987).

### Reverse transcriptase PCR for hMAGI1d detection in clinical samples

Clinical samples were collected as part of an IRB protocol where all patients provided written informed consent (IRB number 2009-274). RNA was extracted using the Ambion Mirvana RNA isolation kit (Applied Biosystems). Following DNaseI treatment (Applied Biosystems), cDNA was prepared using the Superscript III first-strand synthesis kit (Invitrogen, Carlsbad, CA, USA). To validate the specificity of the reaction, SuperScript III RT enzyme was omitted from the reaction (controls). Samples were PCR amplified in a room, separated from the main lab, according to the following protocol: 100 ng cDNA (or control DNA) was mixed with 1X Platinum Taq Hifi buffer (Invitrogen), 2 mM MgSO_4_, 0.8 mM each dNTP (Invitrogen), 1 uM forward primer, 1 uM reverse primer, 2.5 U Gold Taq (Applied Biosystems), 2.5 U Platinum Taq (Invitrogen) in a 50 ul reaction. The amplification protocol used an initial denaturation step of 10 minutes at 94°C, followed by 45 cycles of 30 seconds at 94°C, 30 seconds at 55°C and 90 seconds at 68°C with a final extension at 68°C for 10 minutes (primer sequences are available in supplemental table S2). Products were separated by electrophoresis in a 2% agarose gel and visualized under UV illumination.

### Human cervical cells express a novel variant of MAGI1

To test the association between the presence of a type I PBM, E6 induced PDZ-protein degradation and the cancer phenotype, an assay was designed to quantify the effects of ectopically expressed HPV E6 proteins on the steady state levels of a human PDZ-protein. Attempts to clone the human homologue of the murine MAGI1c, resulted in the cloning of a novel human MAGI1 isoform that was named hMAGI1d. The hMAGI1d cDNA lacks the 15^th^ and 16^th^ exon compared to hMAGI1c (S2 Fig) resulting in a protein in which A901 was fused to E1066 (numbering according to hMAGI1c), which deletes the fourth PDZ domain (residue 979 – 1065) of hMAGI1c (S2B Fig). A search of expressed sequence tag (EST) libraries on the NCBI server (accessed 09/20/2014) identified a match with a cDNA clone from fetal human heart (GenBank# AI130725). That had the same structure we observed in hMAGI1d. To examine the presence of this isoform in cervical tissue, we analyzed RNA extracted from a panel of human cervical cancer tissues by designing a PCR assay that distinguished human MAGI1c from hMAGI1d (S3 Fig). The experiment provided evidence for the co-expression of both hMAGI1c and hMAGI1d in cervical tissue (S2C Fig). The PDZ-1 domain of MAGI1 confers specificity to HPV E6 binding [[1](#_ENREF_1)] thus, lack of the fourth PDZ domain is not expected to interfere with E6 binding and degradation of hMAGI1d.

1. Thomas M, Dasgupta J, Zhang Y, Chen X, Banks L (2008) Analysis of specificity determinants in the interactions of different HPV E6 proteins with their PDZ domain-containing substrates. Virology 376: 371-378.
